# The dark septate endophyte *Phialocephala sphaeroides* suppresses conifer pathogen transcripts and promotes root growth of Norway spruce

**DOI:** 10.1093/treephys/tpac089

**Published:** 2022-07-24

**Authors:** Kai Wang, Zilan Wen, Fred O Asiegbu

**Affiliations:** College of Forestry, Fujian Agriculture and Forestry University, Fuzhou 350002, China; Department of Forest Sciences, University of Helsinki, PO Box 27, Helsinki FIN-00014, Finland; Department of Forest Sciences, University of Helsinki, PO Box 27, Helsinki FIN-00014, Finland; Department of Forest Sciences, University of Helsinki, PO Box 27, Helsinki FIN-00014, Finland

**Keywords:** dark septate endophyte, fungi–fungi–plant interaction, growth promotion, transcriptome

## Abstract

Plant-associated microbes including dark septate endophytes (DSEs) of forest trees play diverse functional roles in host fitness including growth promotion and increased defence. However, little is known about the impact on the fungal transcriptome and metabolites during tripartite interaction involving plant host, endophyte and pathogen. To understand the transcriptional regulation of endophyte and pathogen during co-infection, Norway spruce (*Picea abies*) seedlings were infected with DSE *Phialocephala sphaeroides*, or conifer root-rot pathogen *Heterobasidion parviporum*, or both. *Phialocephala sphaeroides* showed low but stable transcripts abundance (a decrease of 40%) during interaction with Norway spruce and conifer pathogen. By contrast, *H. parviporum* transcripts were significantly reduced (92%) during co-infection. With RNA sequencing analysis, *P. sphaeroides* experienced a shift from cell growth to anti-stress and antagonistic responses, while it repressed the ability of *H. parviporum* to access carbohydrate nutrients by suppressing its carbohydrate/polysaccharide-degrading enzyme machinery. The pathogen on the other hand secreted cysteine peptidase to restrict free growth of *P. sphaeroides*. The expression of both DSE *P. sphaeroides* and pathogen *H. parviporum* genes encoding plant growth promotion products were equally detected in both dual and tripartite interaction systems. This was further supported by the presence of tryptophan-dependent indolic compound in liquid culture of *P. sphaeroides*. Norway spruce and Arabidopsis seedlings treated with *P. sphaeroides* culture filtrate exhibited auxin-like phenotypes, such as enhanced root hairs, and primary root elongation at low concentration but shortened primary root at high concentration. The results suggested that the presence of the endophyte had strong repressive or suppressive effect on *H. parviporum* transcripts encoding genes involved in nutrient acquisition.

## Introduction

All macro-organisms including plants and forest trees are associated with huge and diverse number of microbiota in natural condition. These microbes play various functional roles either positively or negatively on the growth, development and health of their host plants. Microbe–microbe interaction might also positively affect the health of host plants. For instance, many antagonistic microbes are regarded as potential biocontrol agents to suppress plant diseases (Chan et al. 2007, Macarisin  et al. 2010, Liu et al. 2013, Tang et al. 2018, Naik et al. 2019, Eitzen et al. 2021). In natural forest ecosystem, tree-associated fungal endophytes have health beneficial and tree growth promoting effects (Terhonen et al. 2019). Biocontrol microbial agents with plant growth-promotion ability are considered as a safe alternative to chemical control in agriculture and forest environments. Several antagonistic microbes have been applied in practice, such as the application of biocontrol fungus *Phlebiopsis gigantea* (Rotstop^®^) in coniferous forest to control root and butt rot disease caused by *Heterobasidion* species (Pratt et al. 2000, Berglund and Rönnberg 2004, Nicolotti and Gonthier 2005, Oliva et al. 2017).

Antagonistic microbes usually have the capability of competing for nutrients and space, and/or secreting enzymes, antibiotics to suppress plant pathogens (Liu et al. 2013, Naik et al. 2019). For instance, a protein member of glycoside hydrolase family 25 (GH25) of yeast *Moesziomyces bullatus ex Albugo* has been demonstrated for its antagonistic effect against pathogenic oomycete *Albugo laibachii*, contributing to reduced infection in Arabidopsis (Eitzen et al. 2021). A subtilisin-like protease, secreted by biocontrol fungus *Trichoderma harzianum*, displays antifungal activity against multiple phytopathogenic fungi (Yan and Qian 2009, Fan et al. 2014). Moreover, conifer endophyte *Phialocephala scopiformis* is reported to produce anti-insect secondary metabolites (Sumarah and Miller 2009).

A number of plant growth-promoting (PGP) endophytes have been previously described, including bacteria *Bacillus* spp., *Pseudomonas* spp., *Streptomyces* spp., *Burkholderia* spp. (Sun et al. 2009, Kurepin et al. 2015, Afzal et al. 2017, Vurukonda et al. 2018, Wang et al. 2021), filamentous fungi (Alberton et al. 2010, Khan et al. 2012) and some yeasts (Nassar et al. 2005, Nutaratat et al. 2014, Firrincieli et al. 2015). The mechanisms of PGP by microbes include nitrogen fixation, alteration of plant hormone network, production of plant hormones, siderophore, phenazine (Sessitsch et al. 2012, Nutaratat et al. 2014, Naik et al. 2019). Plant root elongation, height and mass increase, yield improvement are also common phenotypic effects of PGP. However, many puzzles related to the mechanism of PGP by beneficial microbe remain to be unravelled.

*Phialocephala* species, the dark septate endophytes (DSEs) that belong to the group of ascomycetous fungi, are widely distributed and associated with forest trees (Kowalski and Kehr 1995, Wilson et al. 2004, Schlegel et al. 2016, Walker et al. 2016). PGP fungi and pathogen/insect antagonistic fungi in this genus have previously been reported (Terhonen et al. 2014, Walker et al. 2016, Narisawa 2017, 2018). The DSE *Phialocephala sphaeroides*, used in this study, was originally isolated from Norway spruce root (Terhonen et al. 2014). PGP and antagonistic effect of *Phialocephala* species have previously been demonstrated (Mandyam and Jumpponen 2005, Alberton et al. 2010, Tellenbach et al. 2013, Terhonen et al. 2014, 2016). Although many PGP microbes and antagonistic microbes have been documented, our knowledge on the interaction mechanisms, especially on how they interact on transcriptomic and proteomic levels, is still limited. The physiological and metabolic changes of PGP microbes and antagonistic microbes are largely unknown. Wen et al. (2022) recently reported that Norway spruce seedlings inoculated with DSE *P. sphaeroides* exhibited PGP effect in promoting primary root elongation. In addition, there was no negative effect on seedlings co-infected with both the endophyte and pathogen *Heterobasidion parviporum*, compared with those infected with pathogen alone. In addition, multiple host transcriptional responses in Ps-inoculated seedlings could have contributed to the root growth promotion (Wen et al. 2022). However, the transcriptomic and metabolic insights from the perspective of the interacting fungi (Ps and Hp) were not evaluated. The aim of this study was to identify transcriptomic and metabolic features associated with spruce growth promotion and pathogen inhibition by the endophyte. Norway spruce seedlings inoculated with Ps-alone (PaPs), Hp-alone (PaHp), and co-infected with Ps and Hp (PaPsHp) were selected for total RNA isolation. Thus, transcripts of both spruce (Wen et al. 2022) and fungi were sequenced. The results supplement the earlier published findings on the host transcriptome (Wen et al. 2022). In the present study, we further analysed the deep sequenced RNA sequencing (RNAseq) data. The fungal transcripts or sequences were mapped to the reference genomes of *P. scopiformis* or *H. parviporum*. Transcriptomic evidences of pathogen suppression and PGP effects by the endophyte Ps were revealed. Moreover, we detected the presence of indolic compounds with auxin activity and PGP effect in the liquid culture of the endophyte.

## Materials and methods

### Seedling inoculation and RNAseq

Norway spruce seedlings that were inoculated with either *P. sphaeroides* (Ps) and/or the pathogen *H. parviporum* (Hp) in co-cultivation were used for total RNA isolation. The total RNAs include the RNAs from Norway spruce, *P. sphaeroides* and/or *H. parviporum*. The co-cultivation, RNA isolation and RNAseq were conducted as previously described (Wen et al. 2022). Briefly, Norway spruce seeds were surface sterilized and germinated on 1% water agar plate for 2 weeks. The DSE *P. sphaeroides* was cultured on malt extract agar (MEA) plate for 1 month. The cellophane membrane containing the DSE mycelium was moved to petri dish. Two-week-old seedlings were transferred to the medium and laid on the hyphae of the DSE for 1 month, before transferring onto rectangular plates (230 × 82 × 18 mm, Radia Industry Co. Ltd, Japan) with sterile peat-based substrate for another month for adaptation. Agar plugs of 17-day-old *H. parviporum* culture were picked to inoculate seedling roots with/without *P. sphaeroides* inoculation. Thus, PaPs seedlings (2.5 months old) were divided into two subgroups: one subgroup was inoculated with *H. parviporum* (PaPsHp), another without Hp (PaPs). Four systems including seedlings without inoculum (Pa), seedlings with *P. sphaeroides* inoculated (PaPs), seedlings with *H. parviporum* inoculated (PaHp), and seedlings with both *P. sphaeroides* and *H. parviporum* inoculated (PaPsHp) were set up with three biological replicates (three to four seedlings per replicate). The whole seedlings (needles, hypocotyl, roots) were collected 1 month post *H. parviporum* inoculation for RNA isolation with cetyl trimethyl ammonium bromide (CTAB) method (Chang et al. 1993). Paired-end (150 bp) transcriptome sequencing with Illumina NovaSeq 6000 platform was conducted by Novogene, UK.

### RNAseq mapping to fungi

As to read mapping to fungal genomes, after checking RNAseq raw data quality with FastQC v0.11.8 (https://www.bioinformatics.babraham.ac.uk/projects/fastqc/) and MultiQC v1.9 (Ewels et al. 2016), SortMeRNA V4.2.0 (Kopylova et al. 2012) and trimmomatic (Bolger et al. 2014) were conducted to remove ribosomal RNA, adaptor and low-quality sequences (SLIDINGWINDOW: 5: 20 MINLEN: 50). The trimmed sequences were mapped into fungal genome *P. scopiformis* DAOMC 229536 (LKNI00000000) (Walker et al. 2016) or *H. parviporum* (assembly ASM299478v1, PDUQ00000000) with alignment tool STAR (Dobin et al. 2013). Aligned sam files were converted into bam files with SAMtools (Li et al. 2009). Mapped transcripts in bam files were counted with HTSeq (Anders et al. 2015).

### RNAseq and statistical analysis

Principal component analysis (PCA) and hierarchical clustering of samples were carried out with DESeq2 (Love et al. 2014) using combined count tables from different samples, which were transformed with variance stabilizing transformation (vst) and were normalized for library size and RNA composition effect. DESeq2 (Love et al. 2014) was applied with adjusted *P*-value 0.05 to produce differentially expressed gene (DEG). DESeq2 normalized values were used for heatmap (scaled by row) and dendrogram with package gplots heatmap.2 (Warnes et al. 2016) in R. For a comparison, DEG was also produced with edgeR (Robinson et al. 2010) with trimmed mean of M-values (TMM) normalization and *P*-value cutoff 0.05. DEG annotations were manually checked to avoid false computational annotation. Gene ontology (GO) enrichment of DEG (DESeq2 method) was conducted using package clusterProfiler 4.0 (Wu et al. 2021), with *P*-value cutoff 0.01 and *P* adjust method Benjamini-Hochberg (BH). Gene-list enrichment tool in KOBAS 3.0 (Bu et al. 2021) was utilized for Kyoto Encyclopedia of Genes and Genomes (KEGG) pathway enrichment of DEGs, with Fisher’s exact test and false discovery rate (FDR) (Benjamini–Hochberg adjusted) <0.05. For DEG analysis of pathogen Hp transcripts, default adjusted *P*-value 0.1 was used as cutoff in DESeq2 analysis.

*Phialocephala scopiformis* mRNAs that mapped the transcripts from PaPs and PaPsHp samples were searched for PGP-related proteins, such as phytohormone biosynthesis enzymes, 1-aminocyclopropane-1-carboxylate (ACC) deaminase, nitrogen fixation, phenazine biosynthesis, non-ribosomal peptide synthase, polyketide synthase, siderophore biosynthesis (Sessitsch et al. 2012, Wang et al. 2021). Blastx was performed to select hits with cutoff *e*-value <1e-70 and bitscores > 200. Transcriptional abundance of those PGP-related genes from PaPs and PaPsHp samples are listed in Tables 2 and 3. Candidate of effector genes and annotations of *H. parviporum* were applied as previously described (Zeng et al. 2018, Wen, Zeng, et al. 2019). The expression of predicted *H. parviporum* effector genes and total read counts were used for heatmap production with DESeq2 package. Raw count of transcripts with less than (≤) 5 in more than (≥) 80% samples were removed. Read counts were normalized and transformed with vst method. The details of DEGs were further investigated with the gene annotation, which were extracted from genome annotation of reference genomes.

Statistical analysis of relative transcript abundance and indolic compound production were conducted by *t*-test in R (version 4.1.2). Data of indolic compound production was plotted as boxplot with package ggplot2 (Wickham 2011) in R.

### Fungal culture, indolic compound production and culture filtrate treatment on root

*Phialocephala sphaeroides* was cultured in MEA plate at 20 °C. For indolic compound production assay, *P. sphaeroides* was cultivated in malt extract (ME) or ME + 0.1% tryptophan liquid culture. Culture supernatant was centrifuged at 12,000 rpm for 2 min, and upper layer was chosen to detect indolic compounds with ME or ME + 0.1% tryptophan as blank reference. Salkowski reagent was chosen to quantify indolic compound production by Ps (Glickmann and Dessaux 1995, Wang et al. 2016), with indole-3-acetic acid (IAA) applied as standard. Spruce seeds were sterilized in hydrogen peroxide solution (Merck, USA) for 10 min and then were washed with clean water. Sterile seeds were germinated in 2% water agar plate at 16 h/8 h light/dark at 20 °C. Two-week-old spruce seedlings with similar sizes were transferred into new water agar plates. Filtered culture supernatant was added alongside the root, with culture medium as control. The root regions were covered with autoclaved filter papers. Growth of primary roots was monitored and recorded 4 weeks post treatment. Sterile Arabidopsis seeds germinated in half Murashige and Skoog (MS) agar plates at 16 h/8 h light/dark at 20 °C. Root tips of 1-week-old Arabidopsis seedlings were treated with filtered culture supernatant (~5 μl per root), with the same volume of 2 μM IAA as positive control and ME + 0.1% tryptophan medium as negative control. Photos of root hair phenotype were taken 24 h post treatment with ZEISS SteREO Discovery.V20 microscope.

## Results

### Heterobasidion parviporum transcripts decreased dramatically in co-infection

**Figure 1.**
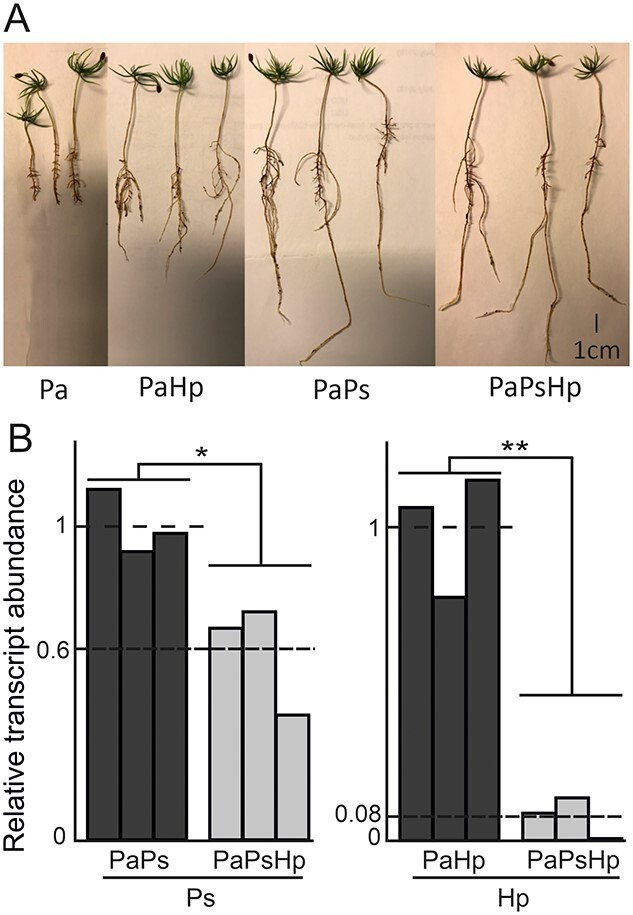
Phenotyping of co-cultivation systems and fungal relative transcription abundance. (A) Norway spruce seedlings were cultured in autoclaved soil with inoculation of pathogen Hp, endophyte Ps and Ps + Hp. Seedlings were kept at growth chamber with 16 h/8 h light/dark at 20 °C. Photos were taken 2 months post inoculation. (B) Relative transcription abundance of Ps from PaPs and PaPsHp samples, as well as of Hp from PaHp and PaPsHp samples. Transcripts were counted as the total transcripts that mapped to the reference genome of *Phialocephala scopiformis* DAOMC 229536 (LKNI00000000) or *Heterobasidion parviporum* (assembly ASM299478v1, PDUQ00000000). Pa, *Picea abies*; Ps, *Phialocephala sphaeroides*; Hp, *Heterobasidion parviporum*. ^*^P < 0.05; ^**^P < 0.01.

Variable effects of fungi inoculation on the growth of spruce seedlings were observed. The endophyte *P. sphaeroides* (Ps) significantly promoted the growth of primary root of spruce, whereas the pathogen *H. parviporum* (Hp) had the opposite effect. Equally, no negative effect on plant growth was observed in seedlings co-infected with endophyte and pathogen (Figure 1A). In order to unravel the activity of Ps and Hp in our co-cultivation system, we mapped and counted the total transcripts of Ps and Hp from spruce seedlings inoculated with Ps-alone (PaPs), Hp-alone (PaHp) and Ps + Hp (PaPsHp). Total mapped transcripts of both Ps and Hp decreased in PaPsHp samples, compared with PaPs or PaHp samples (Figure 1B). The decrease indicated that the presence of Ps had significant repressive or suppressive effect on Hp transcripts. The Ps total mapped reads in PaPsHp samples were 60% of those in PaPs samples. However, the Hp total mapped reads in PaPsHp samples were only 8% of those from PaHp samples (Figure 1B). Total mapping counts and mapping rates are listed in Table 1.

**Table 1:** Number and percentage of uniquely mapped reads of total RNAseq reads that aligned to reference fungal genomes of *Phialocephala scopiformis* or *Heterobasidion parviporum* from sample PaPs, PaHp and PaPsHp.

Mapped reads	Ps1	Ps2	Ps3	Hp1	Hp2	Hp3
PaPs	173,765 (0.17%)	142,893 (0.16%)	151,941 (0.17%)	−	−	−
PaHp	−	−	−	2,021,308 (2.27%)	1,476,638 (1.57%)	2,187,366 (1.96%)
PaPsHp	104,921 (0.12%)	113,034 (0.12%)	61,862 (0.08%)	165,780 (0.19%)	258,842 (0.27%)	11,569 (0.02%)
Ps survival rate	71%	75%	47%	−	−	−
Hp survival rate	−	−	−	8%	17%	1%

Total RNAseq reads contain the transcripts of spruce and fungi. Survival rate refers to the ratio of the percentage of fungal transcripts in co-infected seedlings to the percentage of fungal transcripts in the seedlings infected with individual fungus. Pa, *Picea abies*; Ps, *Phialocephala sphaeroides*; Hp, *Heterobasidion parviporum*.

### Enrichment of GO, KEGG pathway and transcriptomic changes of P. sphaeroides in response to co-infection

**Figure 2.**
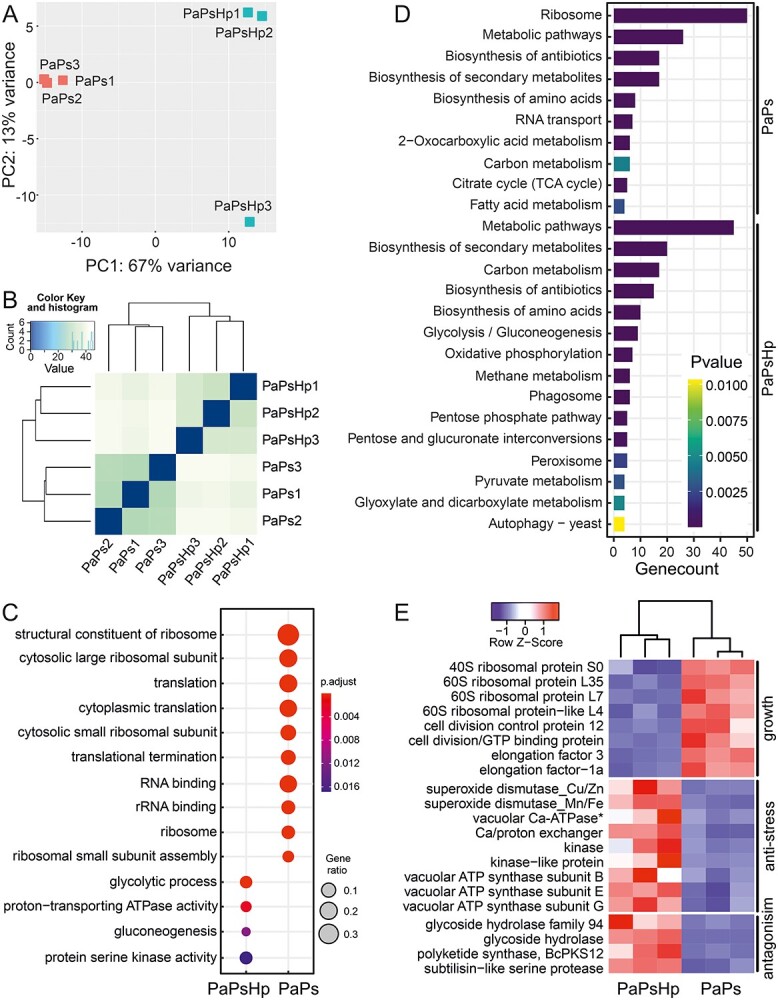
RNAseq profiling of *Phialocephala sphaeroides* in PaPs and PaPsHp samples. (A) PCA plot of total uniquely mapped reads of endophyte *P. sphaeroides* from PaPs and PaPsHp samples. (B) Heatmap of hierarchical clustering of uniquely mapped reads from PaPs and PaPsHp samples. (C) GO enrichment of endophyte *P. sphaeroides* (Ps) from samples PaPsHp and PaPs. List of differential expressed gene (DESeq2 method) was used as input for clusterProfiler 4.0 in R with *P*-value cutoff 0.01 and *P* adjust method BH. (D) KEGG pathway enrichment of *P. sphaeroides* upregulated genes (DESeq2 method) in PaPs and PaPsHp samples (adjusted *P*-value < 0.05, genecount > 3). (E) Heatmap of selected differential expressed genes (DESeq2 method) related to cell growth, stress response and antifungal activity. ^*^: It was annotated as Ca-dependent kinases, which is a mistake from computational annotation. Pa, *Picea abies*; Ps, *Phialocephala sphaeroides*; Hp, *Heterobasidion parviporum*.

We compared the Ps transcriptomic patterns from PaPs and PaPsHp samples. Uniquely mapped reads to Ps reference genome were utilized for further analysis. PCA of PaPs and PaPsHp samples revealed that the two groups were well separated by principal component 1, which accounted for 67% variance (Figure 2A). Hierarchical clustering and heatmap of samples also confirmed the grouping of PaPs and PaPsHp samples (Figure 2B).

Two DEG analysis methods, edgeR and DESeq2, were performed. In summary, there were 74 upregulated genes and 52 downregulated genes in PaPsHp samples compared with those in PaPs samples by edgeR method. With DESeq2, 166 upregulated genes and 146 downregulated genes were detected. The two analysis methods offered similar results and conclusion with DEGs. We observed transcriptomic evidence for the shift from Ps growth to Ps stress response when Hp was added into the cultivation system. GO terms related to glycolytic process, proton-transporting ATPase activity, gluconeogenesis and serine kinase activity in Ps transcriptome were enriched in PaPsHp samples. On the other hand, GO terms about ribosome, RNA binding and translation were enriched in PaPs samples (Figure 2C). Similar to GO enrichment, KEGG pathway enrichment indicated that Ps cell growth-related pathways were enriched in PaPs sample, whereas anti-stress related pathways were enhanced in PaPsHp sample (Figure 2D). This comparison depicted that the Ps translation process was hindered in co-inoculation with Hp. More specifically, genes encoding for 40S-, 60S-ribosomal proteins, elongation factors, cell division control proteins were downregulated, representing a lower speed of Ps growth. By contrast, multiple genes encoding for superoxide dismutase (SOD), vacuolar ATP synthase (V-ATPase), kinase-like proteins and calcium-dependent kinases were upregulated, suggesting an amplified stress response. Transcripts mapped to two SOD genes, copper/zinc SOD and manganese and iron SOD, were upregulated to 5–6-fold in PaPsHp system. Three homologues of V-ATPase genes from Ps were upregulated to 5–8-fold in the co-cultivation system. More interestingly, homologues of genes encoding for inhibitory enzymes, such as glycoside hydrolases, subtilisin-like serine protease and polyketide synthase, were upregulated significantly in PaPsHp samples (Figure 2E).

### Transcriptomic changes of H. parviporum in response to co-infection

**Figure 3.**
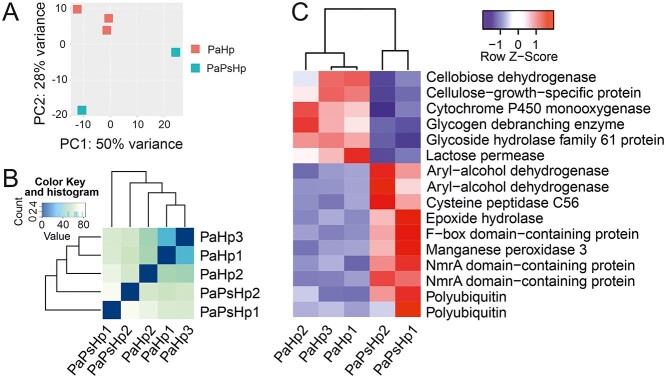
Differential expressed genes of *Heterobasidion parviporum*. (A) PCA plot of total uniquely mapped reads of pathogen *H. parviporum* from PaHp and PaPsHp samples. (B) Heatmap of hierarchical clustering of uniquely mapped reads from PaHp and PaPsHp samples. Sample PaPsHp3 was not included in PCA and hierarchical clustering because of extremely low mapping rate. (C) Heatmap of annotated differential expressed genes from PaHp and PaPsHp samples. Default adjusted *P*-value 0.1 was used as cutoff in DESeq2 analysis. Pa, *Picea abies*; Ps, *Phialocephala sphaeroides*; Hp, *Heterobasidion parviporum*.

Hp transcriptomic patterns from PaHp samples were also compared against PaPsHp samples. Sample PaPsHp3 was excluded in the analysis because of extremely low read counts. PCA plot (PC2 with 28% variance) and hierarchical clustering indicated the separation of the two sample groups (Figure 3A and B). With DESeq2 analysis, 25 genes were considered as DEGs, of which 16 were annotated with known functions (Figure 3C). The expression of genes encoding for carbohydrate/polysaccharide-degrading enzymes (cellobiose dehydrogenase, glycogen debranching enzyme and glycoside hydrolase) and cytochrome P450 monooxygenase were significantly decreased. By contrast, phenolic and epoxide degradation enzyme (Aryl−alcohol dehydrogenase, epoxide hydrolase) genes were upregulated in PaPsHp samples (Figure 3C).

**Figure 4.**
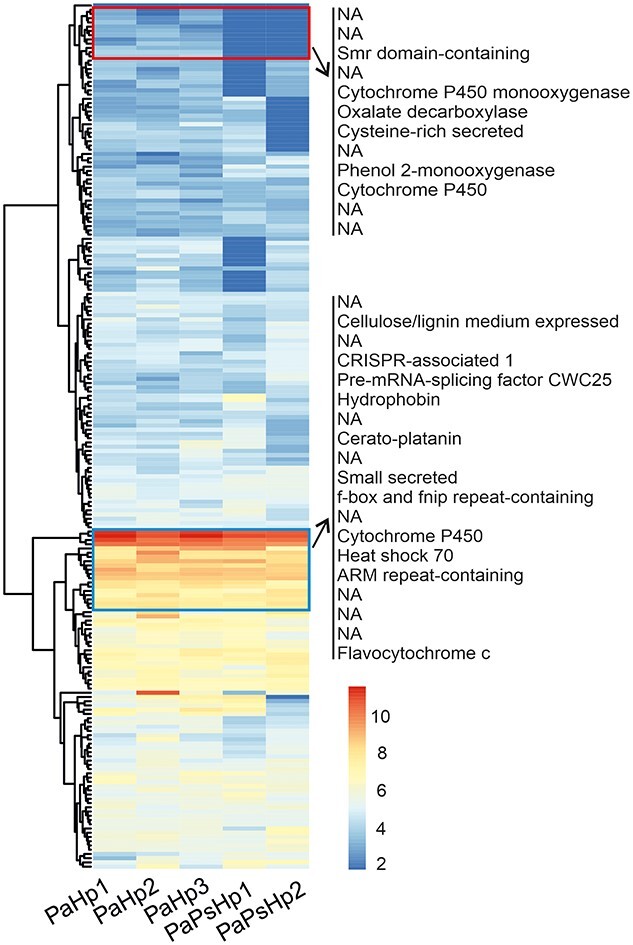
Heatmap of the expression of secreted protein genes of *Heterobasidion parviporum*. The expression of predicted Hp effector genes and total read counts were taken into heatmap production with DESeq2 package. Raw count of transcripts with less than (≤) 5 in more than (≥) 80% samples were removed. Read counts were normalized and transformed with variance stabilizing transformation (vst) method. Genes with lower expression in PaPsHp sample were indicated in red rectangle, and genes with high expression in both PaHp and PaPsHp samples were indicated in blue rectangle. Pa, *Picea abies*; Ps, *Phialocephala sphaeroides*; Hp, *Heterobasidion parviporum*.

In addition, we monitored the expression of Hp effector candidate genes (Wen, Zeng, et al. 2019) in PaHp and PaPsHp samples. Although none of the potential effector gene was detected as DEG with DESeq2 analysis, there was a trend that some clusters of pathogenicity-related genes were expressed at a lower level in PaPsHp samples (Figure 4). Some secreted protein genes with high expression in both PaHp and PaPsHp samples were also identified, which include genes encoding for hydrophobin, cytochrome P450, cerato-platanin and other proteins that might play important roles during the interaction with their host Norway spruce (Figure 4).

### Fungal expression of PGP genes

To understand growth promotion effect by Ps, we investigated the Ps mapped transcripts from both PaPs and PaPsHp samples. Interestingly, transcripts mapped to auxin and cytokinin biosynthesis, polyketide synthase and molecular chaperone HscA genes were discovered (Table 2). Among them, one indole-3-acetaldehyde dehydrogenase (Ald) gene, one polyketide synthase gene and four molecular chaperone HscA genes were highly expressed, which might reflect PGP traits. Notably, the expression of those Ps genes were not negatively affected in PaPsHp samples, which was consistent with the same level of PGP phenotype in PaPs and PaPsHp samples (Figure 1A). Other microbial genes related to PGP, such as ACC deaminase gene, nitrogen fixation nifH, nifD, nifK genes and phenazine biosynthesis genes were not found in transcripts of PaPs or PaPsHp samples. Interestingly, PGP-related genes were detected, with an overall higher diversity and abundance, in the transcripts aligned to pathogen Hp genome. Transcripts mapped to auxin and cytokinin biosynthesis gene were evenly expressed in both PaHp and PaPsHp samples. Similarly, the expression of genes for polyketides and iron–sulphur proteins were also observed from the pathogen Hp (Table 3).

**Table 2:** The transcripts of endophyte *Phialocephala sphaeroides* related to plant growth promotion enzymes discovered from PaPs and PaPsHp samples.

Annotation	Ps gene	PaPs 1	PaPs 2	PaPs 3	PaPsHp 1	PaPsHp 2	PaPsHp 3	%	*e*-value	Bit-score
Hormone										
Indole-3-acetaldehyde dehydrogenase	XM_018205505.1	236	217	180	206	470	292	64	0	657
Cytokinin riboside 5′-monophosphate phosphoribohydrolase	XM_018206012.1	1	1	4	10	4	12	53	9e-73	224
Polyketide										
Polyketide synthase	XM_018218844.1	12	6	11	23	14	37	33	7e-110	387
Iron–sulphur protein										
Molecular chaperone HscA	XM_018205861.1	208	326	185	91	97	74	39	2e-114	358
Molecular chaperone HscA	XM_018209936.1	68	63	42	16	20	27	42	3e-130	395
Molecular chaperone HscA	XM_018210372.1	390	449	404	135	130	128	39	2e-95	306
Molecular chaperone HscA	XM_018214238.1	370	373	181	736	903	253	41	5e-118	365

DESeq2 normalized transcript counts of Ps from different samples were shown. *E*-value <1e-70 and bitscores > 200 were applied in blastx hit selection. Pa, *Picea abies*; Ps, *Phialocephala sphaeroides*; Hp, *Heterobasidion parviporum*; %, amino acid sequence identity.

**Table 3:** The transcripts of pathogen *Heterobasidion parviporum* related to plant growth promotion enzymes discovered from PaHp and PaPsHp samples.

Annotation	Hp gene	PaHp 1	PaHp 2	PaHp 3	PaPsHp 1	PaPsHp 2	%	*e*-value	Bit-score
Hormone									
Amine oxidase	scaffold32.110	46	36	57	78	54	40	4e-160	478
Indole-3-acetamide hydrolase	scaffold1.668	36	47	31	4	57	36	3e-79	256
Indole-3-acetamide hydrolase	scaffold16.14	31	42	19	48	51	40	2e-96	298
IAAld dehydrogenase	scaffold2.297	80	132	93	69	137	34	8e-86	281
IAAld dehydrogenase	scaffold20.70	35	20	33	26	31	36	3e-95	297
IAAld dehydrogenase	scaffold3.523	2,166	2,945	2,240	2,295	2,478	60	0	623
IAAld dehydrogenase	scaffold2.626	80	92	80	117	66	53	0	529
IPyA decarboxylase	scaffold11.216	62	68	50	61	83	51	0	562
Tryptophan aminotransferase	scaffold49.11	55	41	43	39	23	34	3e-85	272
Tryptophan decarboxylase	scaffold3.307	24	15	28	17	17	35	1e-94	295
YUCCA flavin monooxygenase	scaffold16.22	33	12	29	9	26	33	5e-98	311
YUCCA flavin monooxygenase	scaffold2.1024	10	6	10	4	9	40	5e-155	458
YUCCA flavin monooxygenase	scaffold27.43	206	183	213	234	174	43	2e-168	493
YUCCA flavin monooxygenase	scaffold8.64	4	11	2	4	6	34	1e-94	304
Cytokinin dehydrogenase	scaffold1.306	41	27	62	35	54	38	5e-109	336
Cytokinin dehydrogenase	scaffold12.90	37	27	18	35	71	51	2e-175	528
Cytokinin dehydrogenase	scaffold7.26	42	11	48	74	9	50	2e-159	466
Polyketide									
Polyketide synthase	scaffold12.21	27	34	30	52	29	35	4e-152	517
Polyketide synthase	scaffold47.18	63	55	65	48	63	57	4e-90	267
Iron–sulphur protein									
Molecular chaperone HscA	scaffold145.1	22	32	17	35	31	37	5e-102	322
Molecular chaperone HscA	scaffold19.1	80	117	62	135	71	37	5e-104	327
Molecular chaperone HscA	scaffold2.914	135	180	137	139	151	38	1e-102	323
Molecular chaperone HscA	scaffold3.76	237	151	235	187	117	38	3e-112	350
Molecular chaperone HscA	scaffold58.28	109	367	119	108	123	42	6e-127	388
Cysteine desulphurase IscS	scaffold4.267	61	134	56	48	66	56	7e-169	481

DESeq2 normalized transcript counts of Hp from different samples were shown. *E*-value <1e-70 and bitscores > 200 were applied in blastx hit selection. Pa, *Picea abies*; Ps, *Phialocephala sphaeroides*; Hp, *Heterobasidion parviporum*; IAAld, indole-3-acetaldehyde; IPyA, indole-3-pyruvate; %, amino acid sequence identity.

### Indolic compound production and plant growth promotion by P. sphaeroides filtrate

**Figure 5.**
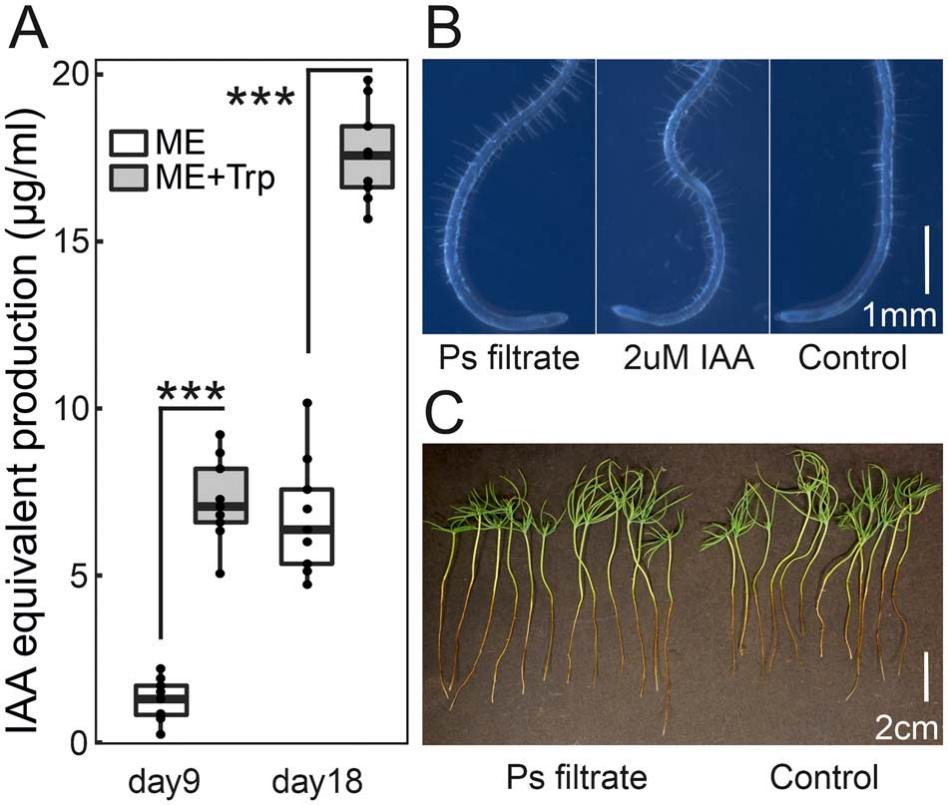
Indolic compounds production and activity by *Phialocephala sphaeroides*. (A) The indolic compounds in *P. sphaeroides* liquid culture were quantified using Salkowski reagent at wavelength 530 nm. A linear model of IAA concentration and OD530 was built as standard for quantification. Two types of growth media (ME, ME + tryptophan) and two time-points (9 day, 18 day) were selected. ME, malt extract; Trp, tryptophan. *T*-test and plotting were performed in R (^***^<0.001). (B) Root hair enrichment of 1-week-old *Arabidopsis thaliana* when treated with filtered filtrate of Ps liquid culture (10× dilution). Arabidopsis root tips treated with Ps medium (ME + tryptophan) was applied as negative control, and Arabidopsis root tips treated with 2 μM IAA as positive control. (C) Root elongation of 2-week-old Norway spruce seedlings when treated with filtered filtrate of Ps liquid culture (10× dilution). Seedlings treated with Ps medium (ME + tryptophan) was applied as control. Ps, *Phialocephala sphaeroides*.

Plant hormone auxin production was mimicked by detection of indolic compounds in Ps liquid culture. Ps produced indolic compounds at 1.3 μg/ml (IAA equivalent) at 9th day in liquid ME medium. The indolic compounds production significantly increased to 7.2 μg/ml at 9th day in liquid culture supplemented with tryptophan (Figure 5A), indicating the presence of tryptophan-dependent auxin synthesis pathways. The accumulation of indolic compounds increased with time, reaching 6.8 and 17.6 μg/ml at 18th day without or with additional supplementation with tryptophan (Figure 5A). To explore endophyte PGP mechanism at metabolic level, we cultured spruce and Arabidopsis seedlings with exposure of the roots to Ps culture supernatant (ME + tryptophan medium, 18 days). Arabidopsis root tips treated with 1/10× filtered Ps culture supernatant displayed root hair enrichment, similar to the phenotype of those with 2 μM IAA treatment, whereas those treated with ME + tryptophan medium exhibited normal root hair phenotype (Figure 5B). With the application of 1/10× filtered Ps culture supernatant on spruce seedlings, the significant elongation of primary root was observed on water agar plate at 4 weeks post treatment (Figure 5C).

## Discussion

There was large variation of Hp survival rates (8, 17 and 1%) in PaPsHp samples. The possible reason could be that interaction system involving three partners (host–endophyte–pathogen) are much more complicated than host–endophyte or host–pathogen dual-interaction systems. The significant reduction of Hp transcripts in co-infection suggests that metabolic activity of Hp was largely restricted or inhibited by the presence of Ps. Many of the affected Hp transcripts were found to be those encoding carbohydrate-degrading enzymes. Ps probably repressed Hp ability to access carbohydrate nutrients by suppressing its carbohydrate/polysaccharide-degrading enzymes machinery as well as its ability to tolerate toxic environment through negative impact on its cytochrome P450. Conversely, the pathogen secreted cysteine peptidase, most probably as an attempt to restrict the free growth of endophyte, which might contribute to 40% reduction in Ps transcript. Interestingly, increased expression of genes encoding phenolic oxidizing enzymes by pathogen might represent a response to enhanced host defences and/or allelopathic phenolic acid analogues secreted by the endophyte. Equally, some downregulated genes in Ps were related to cell division, elongation factor and ribosomal protein synthesis. This lends credence to the idea that growth of Ps was partly negatively affected. Ribosomal proteins are essential for assembly and function of ribosome, which is required for protein synthesis and cell growth (Warner 1999, Wilson and Nierhaus 2005). All the detected homologues of ribosomal protein genes in DEGs were downregulated, indicating deceleration of protein synthesis and cell growth rate. Interestingly, despite of the negative effect on growth of Ps in PaPsHp system at transcriptomic level, Ps still conferred growth fitness benefits on its host.

Several anti-stress genes were upregulated in Ps transcriptome during co-infection with Hp. SODs are key enzymes in reactive oxygen species (ROS) scavenging systems. Fungal SOD plays a role in detoxification and stress tolerance to induced ROS (González-Guerrero et al. 2010, Xie et al. 2010). Thus, upregulation of two homologues of SOD genes in Ps might function as ROS detoxification in co-infection. V-ATPase functions in the acidification of intracellular compartments in both prokaryotic and eukaryotic cells. Previous studies demonstrated a protection role of V-ATPase when yeast cells are under endogenous oxidative stress and biotic stress (Milgrom et al. 2007, Zhang et al. 2008). Many kinases and kinase-like proteins function in defence/stress signalling in plants and microbes (Lee et al. 2016, Yip Delormel and Boudsocq 2019). The upregulation of stress responsive genes further highlights the ability of Ps to deal with the hostile environment.

The changes of Ps transcriptome also provided evidence of suppressive effect of Hp by Ps. GH25 is an antagonistic effector from phyllosphere yeast to inhibit pathogenic oomycete and reduce infection (Eitzen et al. 2021). Upregulation of glycoside hydrolase genes of Ps in PaPsHp system indicated possible antagonistic mechanism. Subtilisin-like serine protease from biocontrol fungi has been demonstrated to possess antifungal activity (Yan and Qian 2009, Fan et al. 2014). Ps might secrete subtilisin-like serine protease as a strategy to suppress the growth of other competing fungi. Although no obvious inhibition zone of Ps against the growth of Hp was observed on MEA medium (Wen et al. 2022), it is most probable that Ps had a repressive or suppressive impact on Hp metabolism and pathogenicity transcripts in PaPsHp system. This also underlies the complex nature of interaction outcome in artificial media versus in natural forest ecosystem.

Downregulation of some carbohydrate usage enzymes of Hp in response to co-infection indicated the reduced capability to use carbohydrates as energy source. Genes of cytochrome P450 enzymes, which are vital for metabolism and adaptation (Chen et al. 2014) and for potential pathogenicity (Mgbeahuruike et al. 2017, Fessner et al. 2021), were downregulated in co-inoculation system. This evidence, together with the lower expression of some candidate effector genes, suggested that the normal growth and metabolism of Hp was disrupted by Ps during co-infection. Surprisingly, Hp genes encoding phenolic oxidizing enzymes were upregulated, indicating activation of an alternative pathway to source for nutrients. It is also likely that Hp might have secreted some inhibitory compounds to counter the effects of Ps. These defences included upregulation of cysteine peptidase of Hp to restrict the growth of other fungi, and lower expression of oxalate decarboxylase effector gene that could lead to accumulation of oxalic acid for antifungal activity (Mäkelä et al. 2010). In addition, we detected highly expressed candidate effector genes that are necessary for Hp growth, including hydrophobin and cytochrome P450 genes, and that for Hp toxicity such as cerato-platanin gene (Chen et al. 2015, Luti et al. 2020) in both groups of samples. The expression of effector HpSSP35.8 gene, which induces cell death and tree defence responses (Wen, Raffaello, et al. 2019), was kept at comparable levels in both groups of samples. These indicated that Hp growth was largely restricted by Ps in this system. Moreover, host priming by Ps might also contribute to Hp inhibition. Host genes related to jasmonic acid (JA) biosynthesis, plant hormone transduction, mitogen-activated protein kinase (MAPK) signalling and Ca^2+^ signalling were upregulated in PaPs samples (Wen et al. 2022). It suggests immunity responses of Norway spruce primed by Ps. As a conclusion, inhibition to Hp in co-infection system was probably due to a combination of direct antagonistic effect by Ps and indirect effect from the host responses primed by Ps.

Transcripts related to PGP were documented in Ps and Hp transcriptomes. Several transcripts encoding for hormone IAA synthesis, cytokinin synthesis, secondary metabolite polyketide synthesis and iron–sulphur protein maturation (Sessitsch et al. 2012, Wang et al. 2021) were detected in all fungi inoculated samples. Indole-3-acetaldehyde (Ald) functions in microbial IAA synthesis to convert indole-3-acetaldehyde (IAAld) to IAA (McClerklin et al. 2018). Ps Ald genes were highly expressed, indicating the active occurrence of IAA biosynthesis by Ps. Moreover, the presence of necrotrophic pathogen Hp did not affect the expression of Ps Ald gene. This transcriptomic pattern was consistent with the phenotype that PGP effect in PaPs and PaPsHp samples were at the same level. One polyketide synthase and four molecular chaperone HscA (S–Fe protein maturation) genes also exhibited similar expression level. The PGP transcripts were also detected from Hp, which might account for the minor growth promotion observed in PaHp samples compared with uninoculated control samples.

PGP was also recorded with treatment of diluted Ps culture filtrate on spruce seedlings in plates. However, with higher concentration of filtrate, the length of primary root growth was decreased, which is similar to the plant phenotype noted with high auxin concentration. Taken together, we concluded that the Ps secreted compounds had similar active auxin role for Norway spruce. Microbial auxin production plays multiple functional roles in plant growth and development (Vorholt 2012, Sukumar et al. 2013, Chanclud and Morel 2016, Lareen et al. 2016). We revealed that Ps indolic compounds production increased with addition of tryptophan, which represented a tryptophan-dependent IAA synthesis. Indolic compounds in Ps culture filtrate had active auxin-like roles, as seen by enhanced root hairs and changes of primary root. However, Ps culture filtrate contains multiple secreted compounds. We could not exclude the possibility of impacts of other metabolites, such as polyketides.

We characterized the mechanisms of PGP and antagonism of Ps at transcriptomic and metabolic level. Other mechanisms, such as competition for space and nutrients, activation of plant defence might also exist simultaneously for pathogen inhibition effect. In addition, pathogen inhibition could also be mediated by host immune responses primed by endophyte Ps. This is the first exploratory study providing insight on the regulatory suppressive effect at transcriptomic level of this endophyte on the pathogen in a tripartite interaction. Future studies may wish to address the functional relevance of the identified genes in the beneficial interaction between the endophyte and its conifer host.

## Data and materials availability

RNAseq raw reads are available in NCBI database (https://www.ncbi.nlm.nih.gov/, Bioproject PRJNA734471) under accession number SRX11066041-SRX11066042, SRX11066044- SRX11066050.
